# Differential expression of WNT4 in testicular and ovarian development in a marsupial

**DOI:** 10.1186/1471-213X-6-44

**Published:** 2006-10-03

**Authors:** Hongshi Yu, Andrew J Pask, Geoffrey Shaw, Marilyn B Renfree

**Affiliations:** 1 Department of Zoology, The University of Melbourne, Victoria 3010, Australia

## Abstract

**Background:**

WNT4 is a key regulator of gonadal differentiation in humans and mice, playing a pivotal role in early embryogenesis. Using a marsupial, the tammar wallaby, in which most gonadal differentiation occurs after birth whilst the young is in the pouch, we show by quantitative PCR during early testicular and ovarian development that WNT4 is differentially expressed ingonads.

**Results:**

Before birth, WNT4 mRNA expression was similar in indifferent gonads of both sexes. After birth, in females WNT4 mRNA dramatically increased during ovarian differentiation, reaching a peak by day 9–13 post partum (pp) when the ovarian cortex and medulla are first distinguishable. WNT4 protein was localised in the ovarian cortex and at the medullary boundary. WNT4 mRNA then steadily decreased to day 49, by which time all the female germ cells have entered meiotic arrest. In males, WNT4 mRNA was down-regulated in testes immediately after birth, coincident with the time that seminiferous cords normally form, and rose gradually after day 8. By day 49, when testicular androgen production normally declines, WNT4 protein was restricted to the Leydig cells.

**Conclusion:**

This is the first localisation of WNT4 protein in developing gonads and is consistent with a role for WNT4 in steroidogenesis. Our data provide strong support for the suggestion that WNT4 not only functions as an anti-testis gene during early development, but is also necessary for later ovarian and testicular function.

## Background

Sexual development provides an ideal model to study organogenesis since the gonads have the bipotential ability to form an ovary or a testis. Wingless-type MMTV integration site family, member 4 (*WNT4*) is a member of WNT gene family, and encodes a cysteine-rich secreted protein. *WNT4 *plays a pivotal role in early embryogenesis, particularly in the formation of the urogenital system [1-4]. After the action of *Sry *in the XY gonad *Wnt4 *expression decreases to undetectable levels in the developing testis, but remains at high levels in the ovary [4]. This led to the initial hypothesis that WNT4 acts as an anti-testis gene, blocking Leydig cell differentiation and steroidogenesis in the developing ovary [4]. Lack of *Wnt4 *results in masculinization of XX mouse embryos [4] and inhibits the migration of endothelial and steroidogenic cells into the developing ovary [5], while over-expression of *Wnt4 *in the developing testis interferes with testicular vascular development and decreases androgen production [5,6]. Sertoli cell differentiation was also compromised in *Wnt4 *mutant testes, demonstrating that *Wnt4 *has specific and distinct roles in both male and female gonadal development [7].

*Wnt4 *appears to regulate *Dax1 *[8,9], a gene believed to antagonize the function of SRY in the developing gonad. *In vitro*, *Dax1 *transcription can be activated by β-catenin, a key signal-transducing protein in the WNT pathway [9]. Besides *Dax1*, *Follistatin *(*Fst*), encoding a TGF-β superfamily binding protein, may also be a downstream component of *Wnt4 *signalling that regulates vascular boundaries and maintains germ cell survival in the ovary [10]. Furthermore, *Wnt4 *and fibroblast growth factor 9 (*FGF9*) act as antagonistic signals to regulate differentiation of the ovary and testis [11]. To date most of our knowledge about the role of Wnt4 in the mammalian gonad has been based on studies only in the mice. In order to determine the role of WNT4 in formation of the mammalian gonad we characterised its expression in a distantly related mammal.

Marsupials give birth after a relatively short gestation to small altricial young that complete their development during a long lactation period attached to a teat, usually in a pouch. The tammar gonadal ridge develops about 6 days before birth, but the gonads remain undifferentiated until after birth [12-14]. Testicular differentiation begins with the formation of seminiferous cords by day 2 post partum. The ovaries, as in all mammals, differentiate after the testis at about day 8 post partum, almost 14 days after the initial development of the gonadal ridge. In contrast to the tammar, the development of the mouse gonad is extremely rapid and there is only 1 day between the formation of the gonadal ridge and the onset of cord formation in males.

Marsupials have the classical mammalian XY sex determining mechanism [15] with a homologue of *SRY *on the Y chromosome [16]. However, the formation of some secondary sexual characteristics, including the scrotum and mammary glands, are under primary genetic control by genes on the X chromosome, and are not dependant on hormones from the testis [14,17,18]. Several key genes in the sex determination and differentiation cascade, *SRY *[19], *SOX3 *[20], *SOX9 *[21], *SF-1 *[22], *DAX1 *[23], *DMRT1 *[24,25], *ATRX/Y *[26,27], *AMH/MIS *[28], have now been cloned and characterized. The endocrine control of male sexual differentiation in the tammar has also been defined [29-35]. Taken together, these data form a primary framework for understanding the evolution of the male sex-determining cascade in marsupials. However, nothing is yet known of the female gonadogenesis pathway in marsupials. This study has therefore characterized WNT4 during early development to gain insight into the onset of ovarian differentiation in a marsupial, and also to determine its expression during the extended period of testicular differentiation.

## Results

### Cloning and characterization of the tammar *WNT4 *Gene

Sequence analysis confirmed that PCR products were derived from the *WNT4 *gene (data not shown). The longest sequence encodes a predicted protein of 351 amino acids.

**Figure 1:**
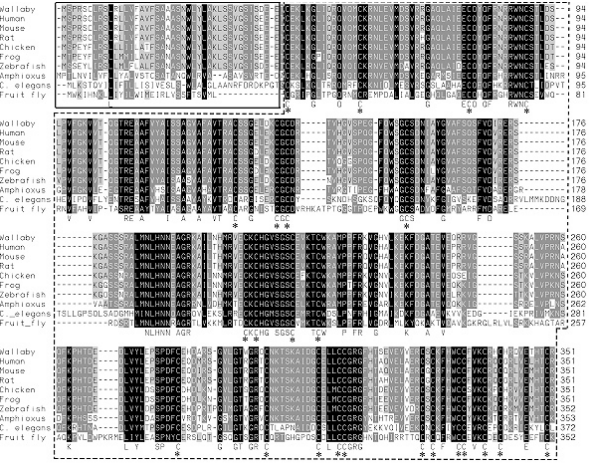
**Amino acid sequence alignment of WNT4 orthologues**. The solid black box indicates the signaling peptide (transmembrane domain) of the WNT4 protein, while the dashed line outlines the WNT gene family domain. Conserved cysteine residues (C) important for secondary structure are marked with an asterisk. Shaded regions indicate residues with shared homology. Numbers indicate residue number.

**Figure 2:**
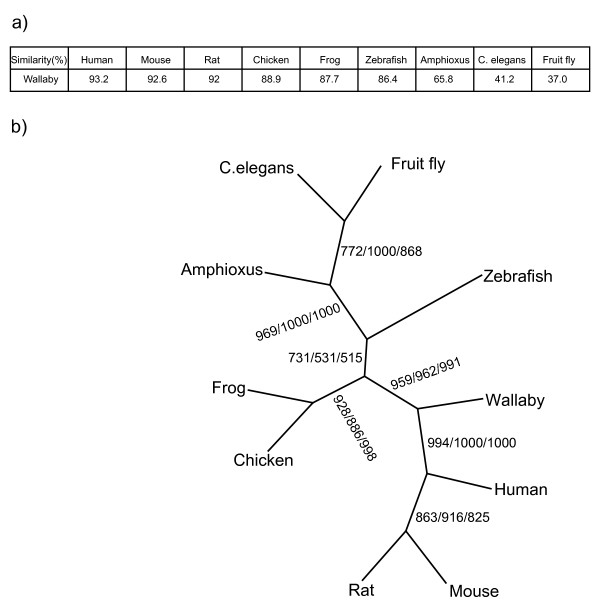
**Similarity and evolution analysis of WNT4 protein**. a) Percentage amino acid similarity of WNT4 between the tammar and other species; b) A phylogenetic tree was constructed with WNT4 protein sequence from the tammar wallaby and other species listed in the methods. Phylogenies were performed with PHYLIP3.63 by bootstrap analysis using Maximum-likelihood analysis (1000 replicates, first values), maximum parsimony (1000 replicates, second values) and neighbour-joining (1000 replicates, third values). Numbers along branches indicate reliability of each branch when run 1000 times.

WNT4 proteins are cysteine rich and highly conserved in all species. The N-terminus contains the transmembrane domains of about 45 amino acids, and other WNT gene family domains, all of which are highly conserved across species (Fig 1). The tammar wallaby WNT4 protein shares 93.2% amino acid similarity with human, 92.6% with mouse, 92.0% with rat, 88.9% with chicken, 87.7% with frog, 86.4% with zebrafish, 65.8% with *Amphioxus *(Fig. 2a). Using the PHYLIP 3.63 program to construct a phylogenetic tree analysis of WNT4 proteins, tammar wallaby WNT4 clusters with eutherian mammals (human, mouse and rat) whereas chicken and frog form another group, zebrafish and *Amphioxus *produce different branches, *C. elegans *and fruit fly create another group distantly related to vertebrates (Fig. 2b).

**Figure 3:**
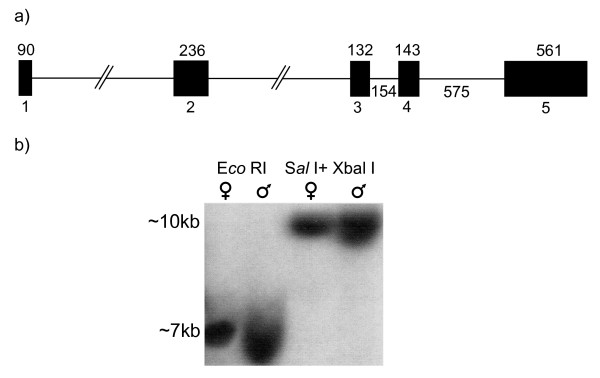
**WNT4 gene structure in the tammar**. a) The tammar wallaby *WNT4 *gene consists of 5 exons. The black blocks represent the exons 1–5, of 90bp, 236bp, 132bp, 143bp and 561bp respectively. The size of intron 1 and 2 are unknown while intron 3 and 4 are 154bp and 575bp accordingly; b) Southern blotting of genomic DNA. Male and female genomic DNA was digested with E*co *RI, S*al *I + X*bal *I, respectively, and hybridized with [α-^32^P]dCTP-labelled tammar *WNT4 *cDNA probe.

Southern blotting analysis of tammar genomic DNA resulted in identical single bands of *WNT4 *in both male and female, indicating there is only a single copy of the *WNT4 *gene in tammar wallaby genome (Fig. 3b). The tammar *WNT4 *gene has 5 exons consistent with that of human, mouse and rat (Fig. 3a). Exon 2, 3 and 4, containing the functional domains, are highly conserved between eutherian mammals and the tammar.

### WNT4 mRNA distribution

**Figure 4:**
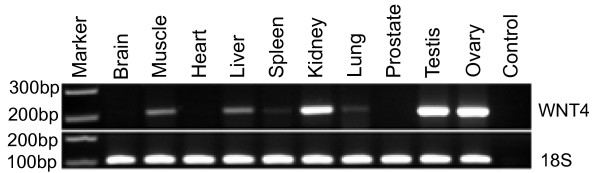
**WNT4 mRNA expression in tammar adult tissues**. Expression of the *WNT4 *gene (209bp) was assessed in a range of adult tissues using the *18S *mRNA (100bp) as a reference. There was very strong expression in the testis, ovary and kidney, moderate expression in muscle and liver, weak expression in spleen and lung, and none in the brain, heart and prostate.

Semi-quantitative RT-PCR was carried out to analyze *WNT4 *gene expression patterns in various adult tissues. *WNT4 *expression was high in the testis, ovary and kidney. Expression was weaker in the muscle and liver, but very low in the spleen and lung and absent in the brain, heart and prostate (Fig. 4).

### Real-time quantitative PCR

**Figure 5:**
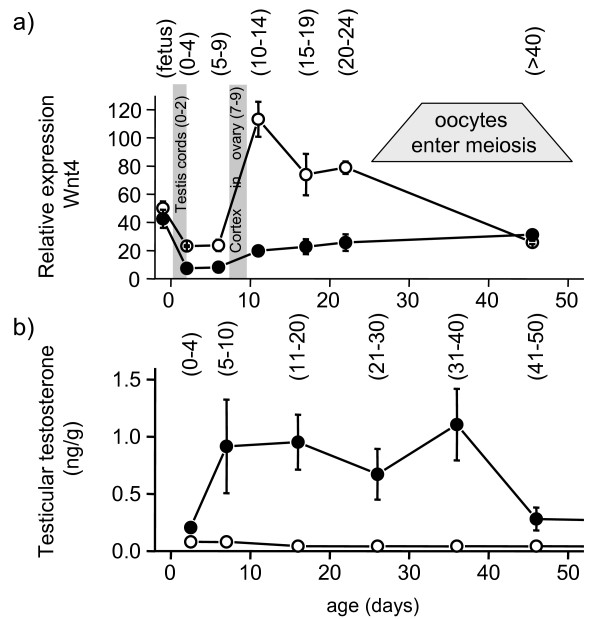
**Quantitative analysis of WNT4 expression and testosterone in developing gonads**. (a) Relative expression of *WNT4 *mRNA against a reference of 18S mRNA (means ± s.e.m) for female (○) and male (●) gonads from the last 3 days of the 26.5 day gestation to day 40–49 post partum. Ages in parentheses indicate actual range of ages in the samples assayed. In females, *WNT4 *expression increased sharply between day 4–8 pp and 9–14 pp coinciding with morphological differentiation of the ovary. Relative expression then declined gradually to days 40–49 pp. In males, *WNT4 *expression fell sharply between fetal stages and day 1–3 pp coinciding with morphological differentiation of the testis. *WNT4 *expression gradually rose from day 9 to reach levels similar to those seen in females at day 40–49 pp. (b) Testicular (●) and ovarian (○) testosterone concentrations (ng/mg) throughout the first 50 days post partum. From [29]

Using real-time quantitative PCR, the fetal gonads of both males and females had equally strong *WNT4 *expression levels (P > 0.3) (Fig. 5a). After sexual differentiation, expression patterns differed between ovary and testis (P < 0.0001). In the presumptive ovary, *WNT4 *expression at day 1–3 and 4–8 post partum was half that in fetuses (P < 0.005). *WNT4 *expression then significantly increased (P < 0.001) to reach a peak at days 9–13 pp. At days 14–24 *WNT4 *remained much higher than in the male, but had decreased by day 41–49 (P < 0.001) to the same level as in the male. In contrast, *WNT4 *was dramatically down-regulated to about 25% of the fetal level in the testis between birth and day 8 pp (P < 0.005). After day 9 pp, the relative expression of testicular *WNT4 *doubled but remained at a much lower level than that of the ovary (P < 0.02 at all stages) until day 41–49 when gonads of both sexes had the indistinguishable levels of *WNT4 *expression (P > 0.15) (Fig. 5a).

### Protein localization in tammar gonads

**Figure 6:**
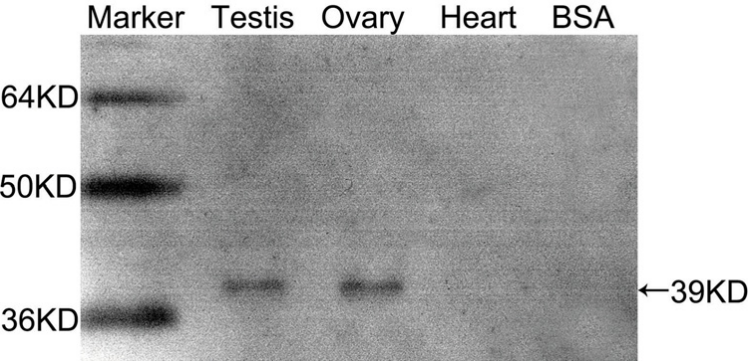
**Western blotting analysis of WNT4**. Testis and ovary protein cross-reacted with the human anti-WNT4 antibody (Abcam), giving a 39 KD band as predicated from the tammar WNT4 sequence. The two negative controls, heart and BSA, did not cross-react.

**Figure 7:**
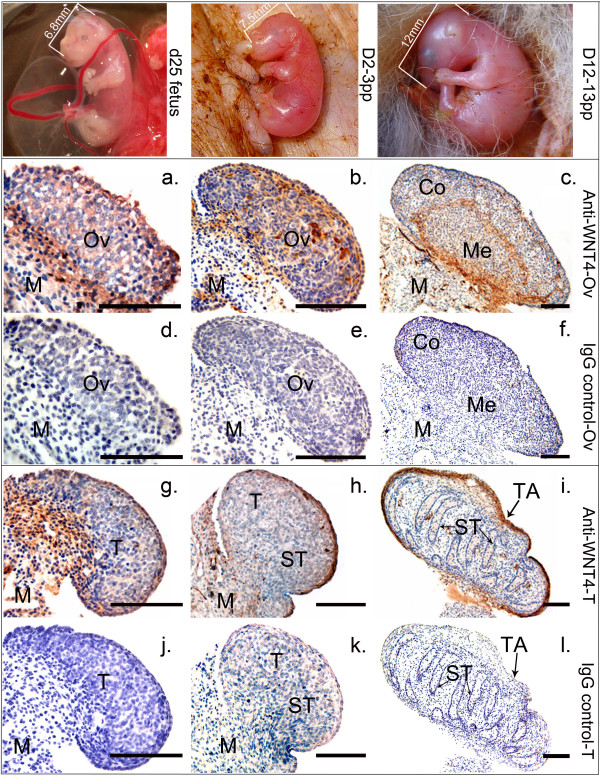
**Immunolocalisation of WNT4 protein during gonadal development in the tammar**. The relative developmental stage of the fetus at each time point examined is shown in the top panels. In fetal gonads one day before birth (d25 of pregnancy), WNT4 protein was distributed broadly in the somatic cells of both females (a) and males (g); from 1–3 days after birth WNT4 stained strongly in the presumptive ovary (b) while it decreased dramatically in the developing testis (h) as the seminiferous cords form. By 12–13 days post partum, WNT4 staining was more specific and clearer. In testis, there was strong WNT4 staining in the Leydig cells and the tunica albuginea (i); there was strong staining in the somatic cells and medulla of the ovary (c). There was no immunoreactivity in any of the negative controls: d, e, f, j, k and l (IgG, pre-immune serum). Co = cortex; M = mesonephroi; Me = medulla; Ov = ovary; ST = seminiferous tubule; T = testis; TA = tunica albuginea. Scale bars= 100 μm

**Figure 8:**
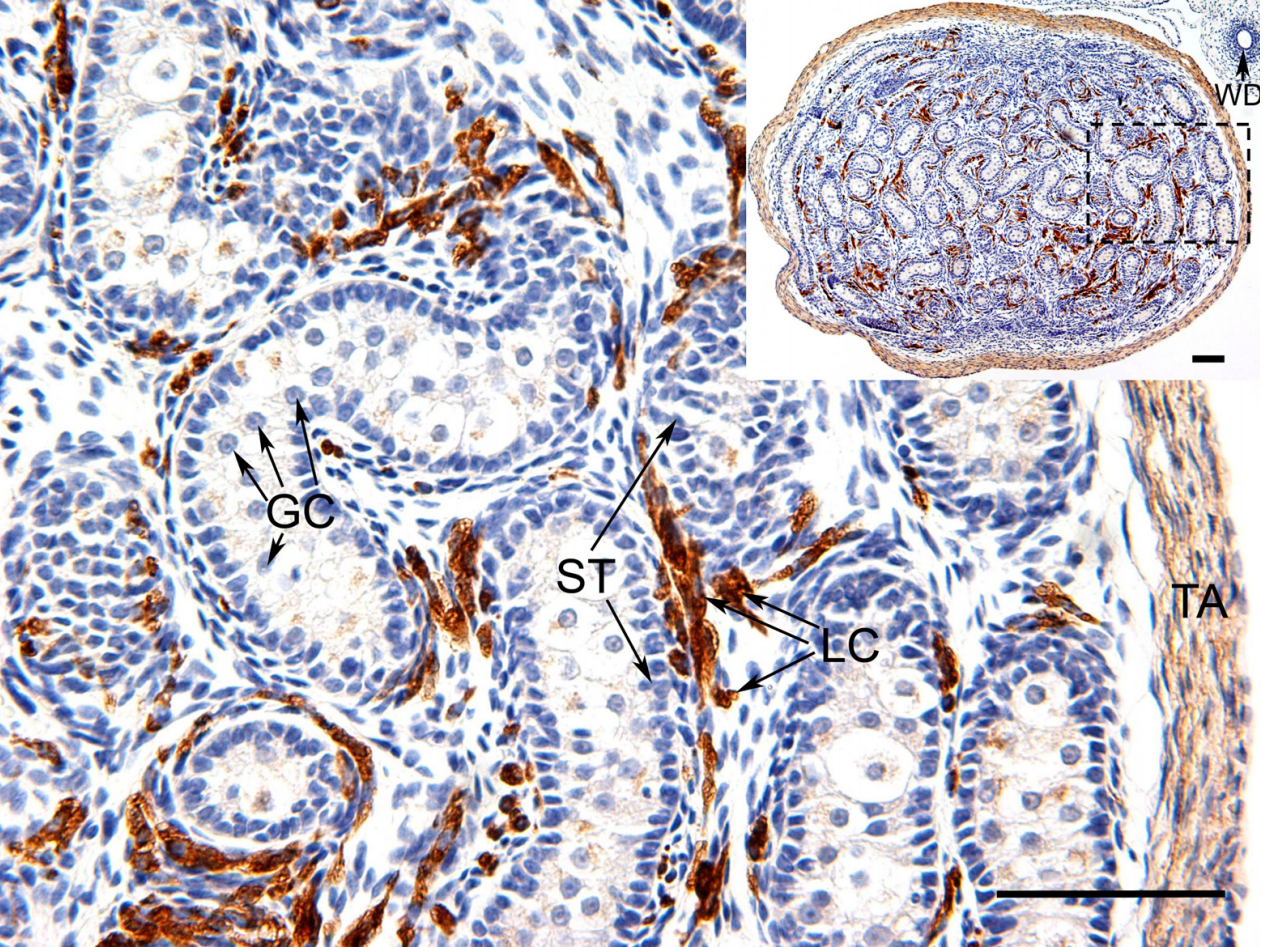
**Immunolocalisation of WNT4 in the testis of the tammar at day 45pp**. WNT4 immunostaining was present in the Leydig cells and the tunica at day 45pp, but it was absent from germ cells and Sertoli cells and Wolffian duct (WD). The dashed square on the low-power image (inset) is the area shown in high-power. Germ cells (GC), Sertoli cells (ST), Leydig cells (LC) and tunica albuginea (TA). Scale bars = 100 μmm.

The human anti-WNT4 antibody (Abcam) had an identical epitope sequence to the tammar WNT4 protein. Western blot analysis detected a single protein of 39KD in each of the testis and ovary extracts, while the two negative controls (heart and BSA) gave no cross-reaction, confirming the specificity of the antibody (Fig 6). Before gonadal differentiation, WNT4 protein was widespread and localised in the somatic cells and surface epithelium of the gonads and mesonephroi of both sexes (Fig 7a, g). In the ovary, after birth WNT4 immunostaining remained strong in the somatic cells (Fig 7b). The WNT4 antibody clearly defined the cords of the rete ovarii and medulla after day 8 (Fig. 7c). Mullerian ducts were positively stained (data not shown). By day 45 post partum, WNT4 staining was strong in the cortical cells of the developing ovary (data not shown). In the testis, shortly after birth when testis differentiation was seen, immunostaining of WNT4 protein decreased (Fig 7h). WNT4 protein stained weakly during testicular differentiation between day 0 and 3, but once initial cord formation was complete, WNT4 expression became stronger in some interstitial cells (Fig 7i). Strong staining was also seen in the tunica albuginea of the developing testis at all stages (Fig 7g, h, and 7i). By about day 45 post partum when germ cells begin to enter mitotic arrest, immunoreactivity in the testis was strong in the interstitium and clearly stained the cytoplasm of most of the putative Leydig cells (Fig 8). However, WNT4 staining was not observed in the Wolffian ducts (Fig 8 inset). In the adult, WNT4 staining was weak in both ovary and testis (data not shown).

## Discussion

We report the first molecular characterization and expression pattern of *WNT4 *in a non-eutherian mammal, the tammar wallaby. This is also the first localization of WNT4 protein in the gonad of any mammal. The greatly extended developmental time of marsupial gonads enabled us to complete the first detailed analysis of the changes in WNT4 expression throughout the whole of this critical developmental period. Since the mouse testis undergoes rapid development, the period of WNT4 down-regulation that we have observed in the tammar testis occurs very rapidly in the mouse and so may have been missed. In the female, *WNT4 *is upregulated during the time of ovarian differentiation, but is down-regulated by the time the XX germ cells have all entered meiosis. In the male, it is down-regulated by the day of birth when testicular differentiation is occurring, but gradually increases by the time XY germ cells have all entered mitotic arrest. The conserved but differing expression patterns of *WNT4 *in the wallaby ovary and testis supports the idea that it has a critical role in gonadal development in both sexes.

The structure and role of *WNT *gene family is highly conserved in vertebrates [36,37], and even in the sea anemone, a species representing the basal group within cnidarians [38]. However, the full-length *WNT4 *gene sequence has only been previously characterized in three mammals, and four non-mammalian vertebrates (Fig. 1). The tammar *WNT4 *gene consists of five exons, exons 2 – 4, the most highly conserved. The tammar *WNT4 *gene is present as a single copy in the genome of both males and females, indicating it is autosomal, as in other mammals (human: Chr 1 [8,39,40], mouse: Chr 4 and rat: Chr 5). WNT4 participates in multiple developmental events during embryogenesis, is broadly expressed in many adult tissues in mice and humans and has also been implicated in adult tissue homeostasis [1-3,7,41,42]. Semi-quantitative RT-PCR expression analyses in adult tammar tissues showed a similar pattern to that of human *WNT4 *consistent with a conserved and wide role for *WNT4 *in tissue homeostasis.

WNT4 also appears to be important for the normal pattern formation and development of both the male and female gonad [7]. In our study, using quantitative RT-PCR and immunohistochemistry, *WNT4 *was expressed at equal levels in the indifferent fetal and neonatal male and female gonads. At this time, protein in the ovary was localized to the somatic cells. Subsequently, *WNT4 *mRNA expression increased significantly to reach a peak at day 9–13, the time that ovarian cortex and medulla can be distinguished. After ovarian differentiation at day 12–13 postpartum, WNT4 expression remained localized in cortex and at the boundary of the cortex and medulla, but by day 45 pp, there was stronger expression in the medulla. The decreased *WNT4 *expression in the ovary to a level similar to that in testis, coincides with the time female germ cells enter meiosis [43].

In the testis, *WNT4 *gene expression was significantly down-regulated immediately after birth during the initial period of testicular differentiation, coinciding with the formation of seminiferous cords [13]. After cord formation, *WNT4 *levels increased slowly after day 8 postpartum and became specific to Leydig cells. By day 49, when secretion of testicular testosterone decreases (Fig. 5b) [32], Leydig cells stained strongly for WNT4. We suggest that once WNT4 reaches a critical threshold level, androgen production diminishes. The low level of immunostaining in the adult testis and ovary is consistent with their increase in steroidogenesis after puberty. This pattern of expression is therefore consistent with the hypothesis that WNT4 blocks initial testis differentiation, because the mRNA concentration is dramatically reduced and the WNT4 protein is localized to the tunica during this period of testicular development.

## Conclusion

The two hypotheses for WNT4 action as an anti-testis gene [4] and as an important factor in testis development [7] are not mutually exclusive. Our data show that WNT4 is down-regulated for a brief period of time when the testis cords are first formed and the cell types of the testis determined, but then becomes up-regulated after day 10 post partum to control later testicular development. In the ovary, WNT4 remains strongly expressed in the gonad consistent with its role in inhibiting early testicular development and promoting later ovarian differentiation. It may also be important for the maintenance of germ cells in both females and males throughout development. Our data support the conclusion that WNT4 functions not only as an anti-testis gene during early mammalian development, but is also necessary for later ovarian and testicular function.

## Methods

### Animals

Tammar wallabies *Macropus eugenii *of Kangaroo Island (South Australia) origin were maintained in open grassy yards in our breeding colony. Fetuses of each sex were collected at various stages of gestation as previously described [44,45]. During the breeding season adult females were checked daily for births (designated day 0 pp). In cases in which the day of birth was uncertain, the age of pouch young was estimated using head length from published growth curves [46]. The sex of pouch young was determined by the presence or absence of scrotal or mammary primordia [17]. All sampling techniques and collection of tissues were approved by The University of Melbourne Animal Experimentation & Ethics Committees. All experimental procedures conformed to Australian National Health and Medical Research Council (1990) guidelines and were approved by Institutional Animal Experimentation Ethics Committees.

### Tissues

Tissues from adult female heart, lung, kidney, liver, spleen, muscle, brain and ovary and from adult male prostate and testis were collected under RNase-free conditions. Gonads were dissected free from the mesonephros from fetuses at day 25, 26 (n = 5 of each sex) and from each day after birth between day 0 and day 49 (Table 1) postpartum pouch young. Tissues for molecular analysis were snap frozen at -80°C until used. Tissues for immunohistochemistry were fixed overnight in 4% paraformaldehyde, washed several times in PBS, and stored in 70% ethanol before paraffin embedding and sectioning at 8 μm.

**Table 1:** Ages and numbers of samples of ovaries and testes analysed by real-time PCR

	Testes		ovaries	
	
	Individuals	total	Individuals	total
Fetuses	d24(1), d25(4), D0(1)	6	d25(3), d26(1), D0(1)	5
D1-3pp	D1(1), D1.5(1), D2(1), D2.5(1),	4	D1.5(1), D2.5(2), D3(1)	4
D4-8pp	D4(1), D6(2), D8(1),	4	D5(1), D7(2), D8(1),	4
D9-13pp	D9(1), D10(1), D11(1), D12(1), D13(1),	5	D9(1), D10(1), D12(1), D13(2),	5
D14-19pp	D14(1), D15(1), D15.5(1), D17.5(2), D18(1),	6	D14(1), D15(1), D16(1), D17.5(1), D18(1), D19(1)	6
D20-24pp	D20(1), D20.5(1), D21(1), D24(2)	5	D20(1), D20.5(1), D21(1) D22(1), D24(1)	6
D41-49pp	D41.5(1), D42(2), D44(1), D49(1)	5	D41(2), D44(1), D45(1), D46(1), D47(2), D47.5(1)	8

### Cloning of tammar WNT4 and determining gene structure

*WNT4 *was initially cloned by RT-PCR using cross species primers designed to conserved regions of the gene (csF1 and csR1; Table 2). The resulting 536bp PCR product was then used to design tammar specific primers for 3' and 5' RACE to fully clone the *WNT4 *transcript (the sequences of all primers are listed on table 2). Only one band was observed for 3' RACE while multiple bands were seen for 5' RACE.

**Table 2:** Primers designed for the analysis of *WNT4 *expression by PCR

***Primers***	***Sequence *(5'→3')**	***Function***
R1	AGCCTGACCATTGGAAGCCCTCT	5'RACE T-PCR
R2	GCCGCACAGAGTCCATCAC	5'RACE
F1	CATCGAGGAGTGCCAGTACCAGTTT	3'RACE
F2	GACGGTGGAACTGCTCGACTCTG	3'RACE T-PCR
SMART IV	AAGCAGTGGTATCAACGCAGAGTGGCCATTACGGCCGGG	5'RACE
CDS III	ATTCTAGAGGCCGAGGCGGCCGACATG-d(T)_30_N_-1_N (N = A, G, C, or T; N_-1 _= A, G, or C)	3'RACE
5' PCR Primer	AAGCAGTGGTATCAACGCAGAGT	5'RACE
qF	GAAACCGACGGTGGAAC	qPCR
qR	AGGAGATGGCATAGACGAA	qPCR
18S F	GATCCATTGGAGGGCAAGTCT	RT-PCR & qPCR
18S R	CCAAGATCCAACTACGAGCTTTTT	RT-PCR & Qpcr
csF1	GTCATCGGTGGGCAGCATCTC	Cross species cloning
csR1	CGTGACACTTGCACTCCACCC	Cross species cloning

F, forward primers in the 5' to 3'; R, reverse primers in the 3' to 5' direction. q, quantitative PCR primers for real time analysis; CDS, Smart IV and 5'PCR were synthesized by SIGAMA (Genosys) according to the manual of the SMART cDNA library construction kit from Clontech.

SMART cDNAs were reverse transcribed from total RNAs from fetuses of wallaby, using the SMART cDNA library construction kit (Clontech, Mountain View, California, USA). 5' RACE was performed using Primer 5' PCR Primer and R1. Owing to an incomplete coding sequence at the 5' end, we designed a new primer R2 according to the first sequence results, and repeated 5' RACE. 3' RACE was performed using primer F1 and CDS III, nested PCR was performed using primer F2 and CDS III. PCR cycling conditions were: 35 cycles, with 30s, 94°C; 40s, 64°C or 56°C, 120s, 72°C, in a 20 μl reaction mix containing 10 mM Tris-HCl, pH 8.3, 1.5 mM MgCl_2_, 50 mM KCl, 200 μM dNTP, 0.2 μM each primer, and 1U Taq DNA polymerase (Promega, Wisconsin, USA).

In order to determine the tammar *WNT4 *gene structure, a *Macropus eugenii *BAC library was screened using *WNT4 *3RACE probe obtained as outlined above. Primers within and spanning intron exon boundaries were designed based on the gene structure of human, mouse and the opossum (deduced from the opossum genome sequence). PCR confirmed the presence of all 4 introns at identical positions to other mammals. We sequenced intron 3 and intron 4 using the BAC plasmid DNA as a template, however, intron 1 and intron 2 were too large to amplify by standard PCR (data not shown).

### Alignment and phylogenetic analysis

WNT4 protein sequences, from tammar (GenBank accession number AY940685), human (NP_110388), mouse (NP_033549), Rat (NP_445854), chicken (NP_990114), frog (P49338), zebrafish (AAA96004), Amphioxus (AAC80431), fruit fly (NP_476810) and C. elegans (NP_493668), was aligned by CLUSTALX 1.83 [47], and edited with GeneDoc)[48]. The phylogenetic tree was constructed with PHYLIP 3.63 program (University of Washington), and viewed with TREE-view 1.6.6.

### Southern blotting hybridization

Genomic DNA was extracted from the liver of the male and female tammar wallabies according to the standard protocol of Sambrook et al. [49], digested with EcoRI and SalI/XbaI, electrophoresed in 0.8% agarose gel, and transferred onto a nitrocellulose filter. Hybridization was performed in ULTRAhyb solution (Ambion Inc., Austin, Texas, USA) with [α-^32^P]dCTP-labeled fragment from primer F2 and CDS III PCR, and autoradiographed.

### Semi-quantitative RT-PCR

Total RNA was isolated from tissues using the RNeasy kit (Qiagen Inc, Valencia, California, USA) or GeneElute kit (Sigma, Castle Hill, NSW, Australia), and the quality and quantity of total RNA were verified by two methods, gel electrophoresis and optical density readings with SmartSpec™ 3000 (BioRad Laboratories Inc., Waltham, Massachusetts, USA). 2 μg of total RNA was DNase-treated with DNA-free (Ambion Inc., Austin, Texas, USA). 1 μg of total RNA was reverse transcribed using SuperScript III (Invitrogen, California, USA).

Primers R1 and F2, spanning the second intron and third intron, were designed from the tammar wallaby *WNT4 *gene structure. PCR was performed in a 50 μl reaction containing 10 mM Tris-HCl, pH 8.3, 1.5 mM MgCl_2_, 50 mM KCl, 200 μM dNTP, 0.2 μM each primer, 1U Taq DNA polymerase (Promega) and first-strand cDNA products. Amplification conditions were: 94°C, 30s; 60°C (18S) or 56°C (*WNT4*), 40s; 72°C, 60s for 26 cycles (18S) or 40 cycles (*WNT4*).

### Real-time quantitative PCR

To obtain further insight into roles of *WNT4 *in sex determination and differentiation, we investigated the *WNT4 *expression profile at different stages of gonadal development by quantitative RT-PCR. Gonads (free of mesonephros) were divided into six groups, each group consisting of 4–8 samples at each stage. Tammar forward primer qF and reverse primer qR were used for real-time PCR and produced a 102 bp fragment. 18S primers produce a 100 bp fragment as internal reference in the quantitative PCR. Primer pair annealing temperature was optimized for real-time PCR on a temperature gradient program. Primer specificity was confirmed by gel electrophoresis and melt curve analysis. To determine the detection range, linearity and real-time PCR amplification efficiency [E = 10^[-1/slope]^] of each primer pair, real-time PCR amplifications were run in triplicate on a 10-fold serial dilution of ovary cDNA and standard curves calculated.

cDNA templates from all stages of gonadal development were prepared as above. Real-time PCR was performed on the DNA Engube Opticon 2 by MJ Research Incorporated (BioRad Laboratories Inc., Waltham, Massachusetts, USA). Each sample was amplified in triplicate in a 20 μl reaction volume using 4 μl cDNA (dilution 10 times before using), 10 μl 2X MasterAmp qPCR SYBR Green Fluorescein Mix (F-400, DyNAmo™ SYRB^® ^Green qPCR kit, Finnzymes Oy, Espoo, Finland), 3 μM of the appropriate forward and reverse primers, and then 3 μl H_2_O to 20 μl final volume. The mixture was incubated at 55°C for 15 minutes, and then at 95°C for 10 minutes to activate the Taq polymerase. This was followed by 40 cycles of denaturation at 95°C for 30 seconds, annealed at 53°C for 20 seconds, extended at 72°C for 35 seconds, and incubated at 76°C for 1 second to read the plate. Finally, a melt curve analysis was constructed from 40 to 95°C.

The initial amplification of PCR product can be dynamically expressed as *X*_*n *_= *X*_0_*(1+*E*)^*n*^, where *X*_*n *_is the number of amplified molecules at cycle *n*, *X*_0 _the initial number of template molecules, *E *the amplification efficiency, and *n *the number of cycles. In fluorescence dye real-time PCR, *X*_*n *_is proportional to the reporter fluorescence *R*, so the above equation can be written as *R*_*n *_= *R*_0_*(1+*E*)^*n*^, where *R*_*n *_is the reporter fluorescence at cycle *n*, and *R*_0 _the initial reporter fluorescence[50]. Therefore, the relative expression of each sample can be calculated by the following formula: ratio = (1+E_Ref_)^CtRef^/(1+E_Target_)^CtTarget^. Values can be assessed to create a graph with the smallest value set as "1" according to the above formula. Comparison of the relative WNT4 expression between males and females at each stage and between stages was achieved using analysis of variance with specified contrasts for individual comparisons (Systat 11, Systat software Inc, Point Richmond, California, USA).

### Western blotting

Developing testis and ovaries (D30-50pp) and two negative adult heart and BSA were collected. Tissues were homogenised in lysis buffer, and extracted total protein. 10 μg of each protein was boiled in sample buffer, electrophoresed on 12% SDS-PAGE and blotted onto Hybond NC membranes (Millipore Corporate, Massachusetts, USA). Goat anti-hWNT4 polyclonal antibody (Abcam, ab15699, Sapphire Bioscience Pty Ltd, Australia) was used as a primary antibody which was diluted 1:750 in 1% BSA/TBS-T. As a secondary antibody, the peroxidase-conjugated anti-goat immunoglobulin was diluted 1:750 in 1% BSA/TBS-T. Signals were visualized by ECL plus™ detection system (Amersham Biosciences UK Limited, Buckinghamshire, UK).

### Immunohistochemistry

Tissue sections (8 μm) were treated with 5% hydrogen peroxide in distilled H_2_O for 10 min to quench endogenous peroxidase activity. Antigen retrieval was achieved by placing slides in boiling sodium citrate buffer (10 mM Sodium Citrate, 0.05% Tween-20, pH 6.0) for 20 min. The primary antibody goat anti-WNT4 polyclonal antibody (Abcam, ab15699) was applied to sections at a 1:200 dilution at 4°C overnight. Signal was amplified using the ABC/HRP kit (DAKO, New South Wales, Australia), visualized with DAB (DAKO), and counterstained with haematoxylin.

## Abbreviations

AMH/MIS, Müllerian-inhibiting substance; ATRX/Y, α-thalassemia and mental retardation associated with the X/Y chromosome; BSA, bovine serum albumin; Dax1, dosage-sensitive sex reversal, adrenal hypoplasia congenita critical region on the X chromosome, gene 1; Dmrt1, Doublesex and mab-3 related transcription factor 1; FGF, fibroblast growth factor; pp, post partum; SF-1, steroidogenic factor 1; SOX, SRY-like HMG box; SRY, sex-determining region of the Y; TGF, Transforming Growth Factor; WNT, wingless related MMTV integration site.

## Authors' contributions

All authors participated in the design of the study. HY and AJP collected the tissue samples. HY, MBR, AJP and GS drafted the manuscript and HY performed all of the experiments. HY, AJP GS and MBR analyzed the results. All authors read, modified and approved the final manuscript.
